# The physiological determinants of drug-induced lysosomal stress resistance

**DOI:** 10.1371/journal.pone.0187627

**Published:** 2017-11-08

**Authors:** Tehetina Woldemichael, Gus R. Rosania

**Affiliations:** 1 Biophysics Program, College of Literature, Science, and the Arts, University of Michigan, Ann Arbor, Michigan, United States of America; 2 Department of Pharmaceutical Sciences, College of Pharmacy, University of Michigan, Ann Arbor, Michigan, United States of America; Purdue University, UNITED STATES

## Abstract

Many weakly basic, lipophilic drugs accumulate in lysosomes and exert complex, pleiotropic effects on organelle structure and function. Thus, modeling how perturbations of lysosomal physiology affect the maintenance of lysosomal ion homeostasis is necessary to elucidate the key factors which determine the toxicological effects of lysosomotropic agents, in a cell-type dependent manner. Accordingly, a physiologically-based mathematical modeling and simulation approach was used to explore the dynamic, multi-parameter phenomenon of lysosomal stress. With this approach, parameters that are either directly involved in lysosomal ion transportation or lysosomal morphology were transiently altered to investigate their downstream effects on lysosomal physiology reflected by the changes they induce in lysosomal pH, chloride, and membrane potential. In addition, combinations of parameters were simultaneously altered to assess which parameter was most critical for recovery of normal lysosomal physiology. Lastly, to explore the relationship between organelle morphology and induced stress, we investigated the effects of parameters controlling organelle geometry on the restoration of normal lysosomal physiology following a transient perturbation. Collectively, our results indicate a key, interdependent role of V-ATPase number and membrane proton permeability in lysosomal stress tolerance. This suggests that the cell-type dependent regulation of V-ATPase subunit expression and turnover, together with the proton permeability properties of the lysosomal membrane, is critical to understand the differential sensitivity or resistance of different cell types to the toxic effects of lysosomotropic drugs.

## Introduction

Alterations in lysosomal structure and function can lead to complex, pathophysiological manifestations in living organisms [1, 2]. For example, mutations which affect proteins mediating lysosomal membrane transport are associated with a variety of inherited diseases and developmental disorders which affect multiple organ systems [3–7]. Physiologically, lysosomal ion homeostasis is maintained by the action of different transmembrane channels and pumps, such as the proton-chloride antiporter known as CLC7 [8], the non-selective cation transporter known as TRPML [9], and the lysosomal proton pump known as vacuolar ATPase (V-ATPase) [10]. *In vivo*, mutations in CLC7 cause osteoporosis and neurodegeneration in mice, and are associated with similar phenotypic effects in humans [11–13]. Similarly, mutations in TRPML are associated with an autosomal-recessive lysosomal storage disease known as MLIV [14–16], whereas mutations in V-ATPase are associated with osteoporosis, renal tubular acidosis, and deafness in humans, and cause similar effects in mice [17–20]. Furthermore, perturbations in vesicular trafficking which affect ion transport functions can adversely affect cellular function which leads to muscle degeneration [21].

Like mutations in proteins affect the ion transport properties of lysosomal membranes, many drugs accumulate in lysosomes or interfere with lysosomal ion transport mechanisms, which lead to alterations in lysosomal structure and function. At the cellular level, the effects of drug accumulation often resemble that of genetic mutations which affect lysosomal ion homeostasis, such as lysosomal size expansion [22] and changes in organelle morphology [23]. Furthermore, *in vitro* experiments have revealed that the accumulation of lipophilic, weakly basic drugs in lysosomes can affect lysosomal pH [23], membrane potential [1], organelle morphology [23], and changes in transmembrane ion permeability [24–26]. Therefore, it is possible that drug-induced lysosomal stress may be manifested as idiosyncratic drug side effects, which include increased predisposition to microbial infections [27], osteoporosis [28, 29], and neurodegenerative diseases; such as Alzheimer’s disease, Huntington’s disease, and Parkinson’s diseases [11, 30, 31].

Interestingly, cells upregulate the expression of genes that allow lysosomes to recover normal physiological function following the disruption of physiological ion homeostasis [32]. At the transcriptional level, the transcription factor EB (TFEB) mediates lysosomal stress pathways [33] by upregulating the expression of lysosomal genes, such as V-ATPase [34] and TRPML1 [33]. In mice, TFEB reverses expanded lysosomes by upregulating genes that directly or indirectly re-establish lysosomal ion homeostasis [33, 35, 36]. As a regulator of lysosomal biogenesis and stress tolerance, TFEB not only affects ion transport functions and membrane trafficking [37, 38], but its activation has also been associated with cells’ increased resistance to physiological perturbations induced by lysosomotropic drugs [39].

In order to further understand how lysosomotropic drugs may affect lysosomal ion homeostasis, a physiologically-based, mathematical modeling approach was utilized to shed light on key parameters which affect recovery from transient perturbations in lysosomal ion regulation. This approach was deemed necessary because pharmacological agents that accumulate in lysosomes can exert multiple effects on the molecular mechanisms that influence lysosomal pH, membrane potential, and chloride transport. These include inhibitors of the V-ATPase, such as Bafilomycin A, Concomycin, Salicylihalamide A, and Archazolid [40, 41], chloride channel blockers, such as Cystic fibrosis transmembrane regulator (CFTR) inhibitors, which include Glibenclamide and Niflumic acid, and ClC channel inhibitor known as Lubiprostone [42], as well as other conditions that affect lysosomal morphology [43, 44] and membrane permeability [45–49]. Upon simulating transient perturbations, parameter sensitivity analysis was used to reveal the most likely mechanistic determinants of the cell’s ability to restore and maintain lysosomal ion homeostasis following exposure to one or more drug-induced lysosomal stresses, thereby providing important theoretical insights into the mechanistic determinants of drug-induced lysosomal stress and stress tolerance.

## Methods

### Model parameterization

**Fig 1:**
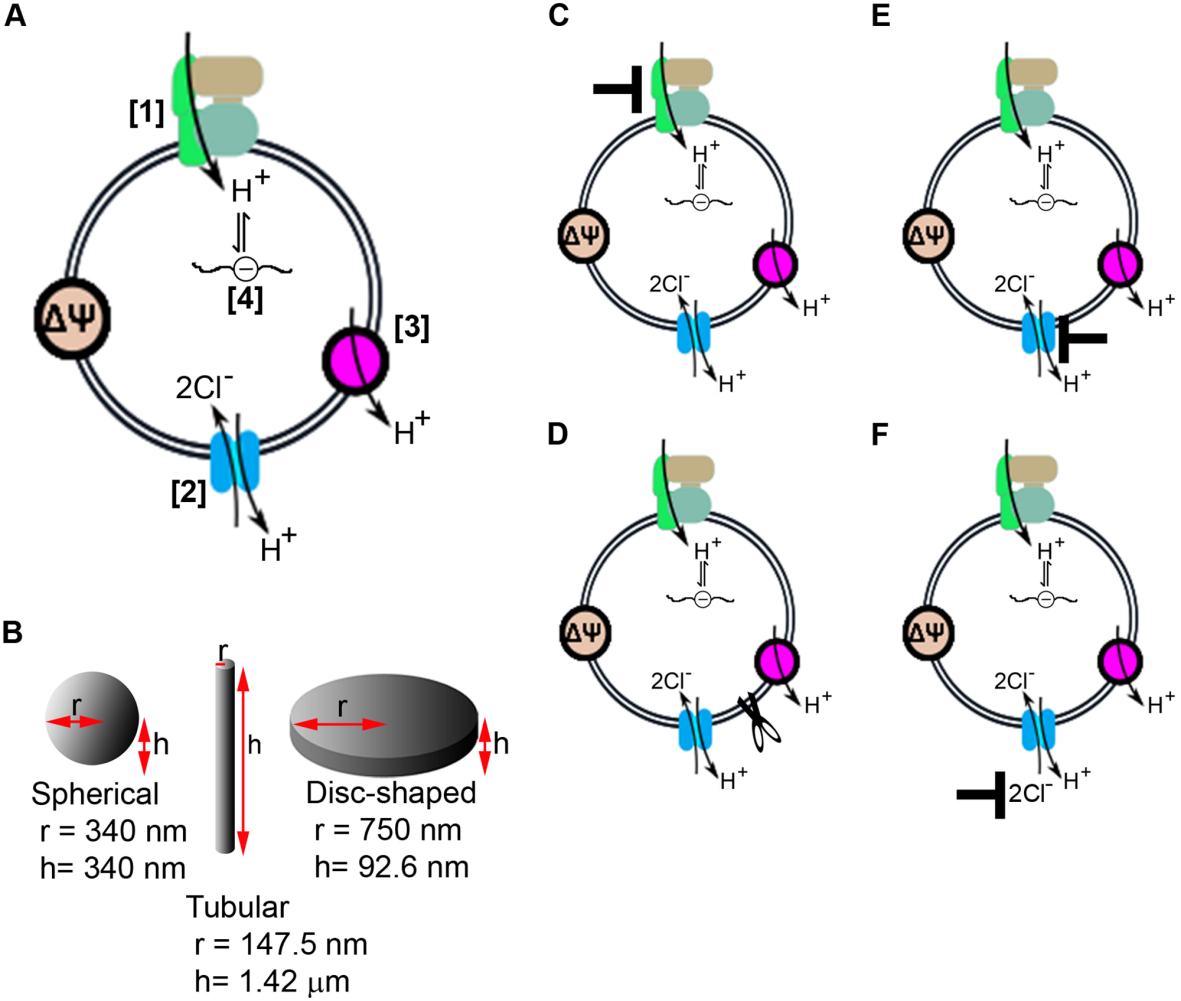
Modeling the effects of drugs on lysosomal ion homeostasis. We utilized an established physiological framework to model lysosomal ion transport (A). Accordingly, the V-ATPase actively pumps protons into the lysosome (1) while the proton-chloride antiporter CLC7 dissipates the ensuing increase in membrane potential by coupling the efflux of protons with the influx of chloride (2). Protons escape the lysosomes through diffusion across the lysosomal membrane (3), and protons in the lysosome are sequestered through the buffering capacity of resident lysosomal components (4). Free protons that accumulate in the lysosomal lumen contribute to the decrease in lysosomal pH. Based on this mechanism, the effect of drugs was captured by modeling different lysosomal shapes and volumes (B). Concomitantly, the drug-dependent inhibition of V-ATPase function was modeled by varying the proton pumping activity (C); the drug-dependent change in membrane proton permeability was modeled by varying the proton leak from the lysosome (D); and, the drug-dependent perturbation of membrane potential regulation was modeled by inhibiting CLC7 (E) or decreasing cytoplasmic chloride (F).

An established, systems-based mechanistic model of lysosomal ion transport, which is comprised of differential equations that capture the transmembrane transport properties of ions and water across the lysosomal membrane [8, 50] was used to simulate the physiological consequences of drug-induced lysosomal stress (Fig 1).

For the purpose of studying the lysosomal stress response, the model (S1 Text) contained 22 parameters. Six out of the 22 parameters were varied to capture the effects of drugs on lysosomal structure and function, whereas the remaining sixteen were fixed (Table 1). Fixed parameters were set to published values, which give rise to physiological lysosomal function [50, 51] and are referred to as “baseline input values”. These parameters include lysosomal radius of 340 nm with volume and surface area corresponding to a spherical lysosomal vesicle, which was obtained from electron microscopy data [52], and the associated organellar ions and ion transporters, which include 300 V-ATPase molecules per lysosome, which was estimated from microscope data analysis and wet lab experimental data fitting [50, 53, 54], membrane proton permeability of 6x10^-5^ cm/s, which was estimated from wet lab experimental data fitting [50], cytoplasmic chloride concentration of 10 mM [55, 56], and initial lysosomal ion concentrations estimated to equal that of extracellular ions [56]. A lysosome with the entire model parameters set to baseline input values is referred to as an “unperturbed lysosome”.

**Table 1:** Model parameters.

Symbol	Description	Baseline Input Value	Range of Input Value	Units
pH_C_	Cytosolic pH	7.2	Fixed	pH unit
pH_L_	Luminal pH	7.4	Fixed	pH unit
[Cl^-^]_C_	Cytosolic chloride concentration	10	1x10^-5^–10	mM
[Cl^-^]_L_	Luminal chloride concentration	110	Fixed	mM
[Na^+^]_L_	Luminal sodium concentration	145	Fixed	mM
[K^+^]_L_	Luminal potassium concentration	5	Fixed	mM
[H^+^]_L_	Luminal proton concentration	0	Fixed	mM
P_H_^+^	Membrane proton permeability	6x10^-5^	1.38x10^-7^–6	cm/s
V	Lysosomal volume	1.65x10^-16^	2.88x10^-17^–1.65x10^-16^	L
S	Lysosomal surface area	1.45x10^-8^	1.45x10^-8^–6.28x10^-6^	cm^2^
C’	Specific bilayer capacitance	1	Fixed	μFarad/ cm^2^
β	Buffering capacity	40	Fixed	mM/pH
N_VATP_	V-ATPase number	300	1x10^-4^–1.3x10^5^	
N_ClC7_	CLC7 number	5000	1x10-4–5000	
Ψ_out_ *	Outer surface potential	-50	Fixed	mV
Ψ_in_ *	Inner surface potential	0	Fixed	mV
CLC_Cl	CLC7 Cl^-^ stoichiometry	2	Fixed	
CLC_H	CLC7 H^+^ stoichiometry	1	Fixed	
R	Gas constant	8.314	Fixed	J.K^-1^.mol^-1^
T	Absolute temperature	0	Fixed	Kelvin
F	Faraday's constant	96485	Fixed	J/volt
N_av_	Avogadro's number	6.02x10^23^	Fixed	molecules/mol

Model parameters used to simulate various lysosomal stresses and stress tolerance mechanism. Baseline input values are literature values [52–56] representing physiological lysosomes and are in agreement with previously published models [50, 51].

* estimated intrinsic surface potentials for inner, Ψ_in_, and outer, Ψ_out_, leaflets of the lysosomal membrane accounted for when modeling membrane transporter mediated dynamic lysosomal and cytoplasmic ion concentrations at the surface [51].

Adjustable parameters are those that were varied from their respective baseline input values in order to simulate the effects of lysosomal stressors. Accordingly, lysosomes that are modeled by changing one or multiple of these adjustable parameters are referred to as “perturbed lysosomes”. Moreover, the lysosomal ion stressors reported here are associated with stressors inducing variations in lysosomal membrane proton permeability, cytoplasmic chloride concentration, and V-ATPase and CLC7 molecules per lysosome, whereas the lysosomal morphology stressors are associated with stressors inducing variations in lysosomal surface area and volume.

### Simulating drug induced changes in lysosomal morphology

To study how drug-induced changes in lysosomal morphology affect ion homeostasis, simulations were performed in lysosomes of different surface areas and volumes. First, lysosomes were modeled as perfect spheres. Assuming there are approximately around 100 lysosomes in a cell, which occupy 1% of cellular volume, the volume of a single lysosome was set to 1.65x10^-16^ L. For a spherical vesicle, this volume corresponds to a lysosomal radius of 0.34 μm and a surface area of 1.45x10^-8^ cm^2^.

For comparison, tubular lysosomes (Fig 1B) (radius = 40 nm—270 nm, height = 585 nm—5.73 μm) were modeled using a range of volumes (2.88x10^-17^ L-1.34x10^-16^ L). The dimensional relationship between the tubular radius and height, at constant lysosomal surface area of 1.45x10^-8^ cm^2^ (equivalent to the surface area of a spherical lysosome (radius = 0.34 μm), as previously mentioned in our study of spherical lysosomes), was calculated using cylindrical equation (V = πr^2^h, where r is radius and h is height). These morphologies are consistent with measurements in other publications [44, 57].

Next, to mimic disc-shaped lysosomes as have been reported in different cell types under other biologically relevant conditions [58–60], lysosomes (radius = 422 nm-10 μm, height = 0.5 nm-294.9 nm) were modeled using a rage of lysosomal surface area (1.90x10^-8^ cm^2^–6.28x10^-6^ cm^2^). The dimensional relationship between the disc-shaped lysosomal radius and height, at constant lysosomal volume of 1.65x10^-16^ L (equivalent to the volume of a spherical lysosome, 0.34 μm in radius), was calculated using cylindrical equation (S = 2πr^2^ + 2πrh, where r is radius and h is height).

### Simulating stress tolerance following drug induced changes in lysosomal morphology

To understand how lysosomes possessing different structural and functional characteristics may respond differently to drugs, the number of V-ATPase and membrane proton permeability -were individually varied. When studying the effects of these two particular lysosomal parameters on morphologically altered or otherwise stressed lysosome, we referred to them as “stress tolerance inducers”. Thus, the number of V-ATPase molecules per lysosome was increased from physiological baseline input value of 300 to 1.3x10^5^ (resulting in 13 data generating points), while proton permeability was decreased from physiological baseline input value of 6x10^-5^ cm/s to 1.38x10^-7^ cm/s (resulting in 9 data generating points). These ranges of input values for both V-ATPase number and proton permeability allowed us to quantitatively compare and contrast the relationship of lysosomal surface area to the number of V-ATPase molecules and membrane proton permeability per lysosome. For example, we chose different fold increments in lysosomal surface area (say, 20- or 400-fold) and one simulation at a time set the corresponding lysosomal surface area as an input. So, in the case of a lysosome with a 20-fold lysosomal surface area expansion, we set the surface area to 2.9x10^-7^ cm^2^. Then, to study the effect of the number of V-ATPase molecules on this expanded lysosome, we performed parametric simulation for the V-ATPase number ranging 0 to 6000, which corresponds to 0 to 20-fold increment in V-ATPase number per lysosome.

### Simulating drug induced lysosomal stress

Parametric simulations were performed to study the effects of the following four drug-induced stresses on lysosomal physiology: V-ATPase inhibition, CLC7 inhibition, lysosomal membrane permeabilization, and decreased membrane potential dissipation based on lower cytoplasmic chloride concentration (Fig 1). More specifically, membrane proton permeability was varied from physiological baseline input value of 6x10^-5^ cm/s to 6 cm/s (resulting in 8 data generating points), the number of V-ATPase molecules per lysosome was varied from 0 to physiological value of 300 (resulting in 10 data generating points), the number of CLC7 molecules per lysosome was varied from 0 to physiological value of 5000 (resulting in 25 data generating points), and the cytoplasmic chloride concentration was varied from 0 to physiological value of 10 mM (resulting in 7–16 data generating points).

Using the aforementioned ranges of the adjustable lysosomal parameters, the corresponding lysosomal parameter inhibition range 0 to 100% was calculated for cytoplasmic chloride, V-ATPase and CLC7 number; where 0% represents no change from respective physiological baseline input value, and 100% represents the input value set to ~zero. The inhibition range was calculated as follows by comparing the corresponding input value (Adjusted Input Value) from the aforementioned given range with its respective physiological input value (Baseline Input Value):

$$\%\;\text{Inhibition}=\frac{\text{Baseline}\;\text{Input}\;\text{Value}-\;\text{Adjusted}\;\text{Input}\;\text{Value}}{\text{Baseline}\;\text{Input}\;\text{Value}}\times 100\%$$ (1)

### Simulating individual lysosomal ion stressors

The effects of the lysosomal ion stressors were individually studied in spherical and different sized tubular lysosomes by performing parametric simulation of the four parameters mentioned in the previous subsection (using the same ranges of input values with corresponding intervals) while setting the lysosomal volume and surface area input values to correspond to either a spherical or tubular lysosomal geometry, as indicated in the earlier subsection. For example, in the case of introducing CLC7 inhibitor to a spherical lysosome, lysosomal volume and surface area were set to 1.65x10^-16^ L and 1.45x10^-8^ cm^2^, respectively. Then, the CLC7 number was varied from 0 to 5000, as previously indicated. Similar approach was applied when studying stressor effect in tubular lysosomes except the input value for lysosomal volume was varied from 2.88x10^-17^ L to 1.65x10^-16^ L (resulting in 4 data generating points), while fixing lysosomal surface area at 1.45x10^-8^ cm^2^.

### Simulating combinations of lysosomal ion stressors

The effects of the various combinations of the aforementioned lysosomal ion stressors were studied in spherical, tubular, and disc-shaped lysosomes. The stressor combinations included V-ATPase inhibition-CLC7 inhibition, V-ATPase inhibition-Cytoplasmic Cl^-^ depletion, V-ATPase inhibition-membrane proton permeabilization, CLC7 inhibition-Cytoplasmic Cl^-^ depletion, CLC7 inhibition-membrane proton permeabilization, Cytoplasmic Cl^-^ depletion-membrane proton permeabilization. The ranges of values, with the associated specific or arbitrarily chosen intervals for each parameter mentioned in the previous sections were applied here as well. For example, to simulate the administration of V-ATPase inhibition-CLC7 inhibition to a spherical lysosome, lysosomal volume and surface area input values were set to 1.65x10^-16^ L and 1.45x10^-8^ cm^2^, respectively to adjust the lysosomal geometry as a sphere. Then, the CLC7 number was varied from 0 to 5000, whereas, the V-ATPase number was, one simulation at a time, manually set to a value within the range 0 to 300.

### Calculating the effects of lysosomal stressors and tolerance on lysosomal physiology

Time-plot simulations were performed to obtain physiological final lysosomal pH, Cl^-^, and membrane potential readout values for an unperturbed lysosome, where all of the parameters are set to physiological baseline values. These readout values, which from hereon we refer to as “physiological baseline readout values”, were specifically chosen as they are direct indicators of lysosomal ion homeostasis and physiology.

Similarly, when performing each of the aforementioned parametric lysosomal stress and stress tolerance simulations, final lysosomal pH, Cl^-^, and membrane potential variables were chosen as readouts as a function of either a specific lysosomal stressor or stress tolerance inducer in order to generate two-dimensional data. Then, the 2D dataset was exported to Microsoft Excel for further analysis. The final lysosomal pH, Cl^-^, and membrane potential values were subtracted from their respective physiological baseline readout values mentioned earlier in order to determine the net effect of the lysosomal stressor or the stress tolerance inducer on lysosomal physiology based on the changes in lysosomal pH, Cl^-^, and membrane potential.

### Confirmation of steady state and mass balance

For all of the aforementioned simulations, the final readout values were confirmed that they were steady state values by performing the simulations for > 24 hours (simulation time). Furthermore, we confirmed that mass balance was attained for conditions where the thermodynamic limit of V-ATPase proton pump (up to 4.6 pH unit gradient) [61] was maintained across the lysosomal membrane.

### 3D data visualization

Multiple individual 2D datasets associated with the aforementioned parametric simulations performed to generate simultaneous inhibitions of various lysosomal parameters were obtained. The datasets associated with each simultaneous inhibitions of lysosomal parameters were exported from Berkeley Madonna and compiled into three separate matrices in an excel spreadsheet, such that the first rows and columns of the matrix correspond to the two parameters simultaneously varied in the model simulations to obtain the final lysosomal readout values (lysosomal pH, Cl^-^, and membrane potential), where each makes up the rest of the rows and columns of a single matrix. 3D surface plot of the matrix was generated using Sigmaplot^®^.

## Results

Using a physiologically-based, mechanistic mathematical model of lysosomal ion transport regulation, we performed computational simulations to reveal how drug-induced variations in one or more ion transport mechanisms influenced lysosomal physiology, as captured by lysosomal pH, Cl^-^, and membrane potential. To facilitate interpretation of these results, we separately considered lysosomal ion stressors and lysosomal morphology stressors. The earlier directly perturb lysosomal ion transportation, while the latter directly perturb lysosomal morphology. As elaborated in the following subsections, the effects of the aforementioned lysosomal stressors on lysosomal physiology were considered in the context of 1) biologically-determined variations in lysosomal morphology, 2) drug induced changes in lysosomal volume and surface area, 3) changes in lysosomal volume and surface area as may happen during endocytosis or exocytosis.

### The effects of individual alteration of lysosomal proton and chloride transportation on lysosomal physiology

**Fig 2:**
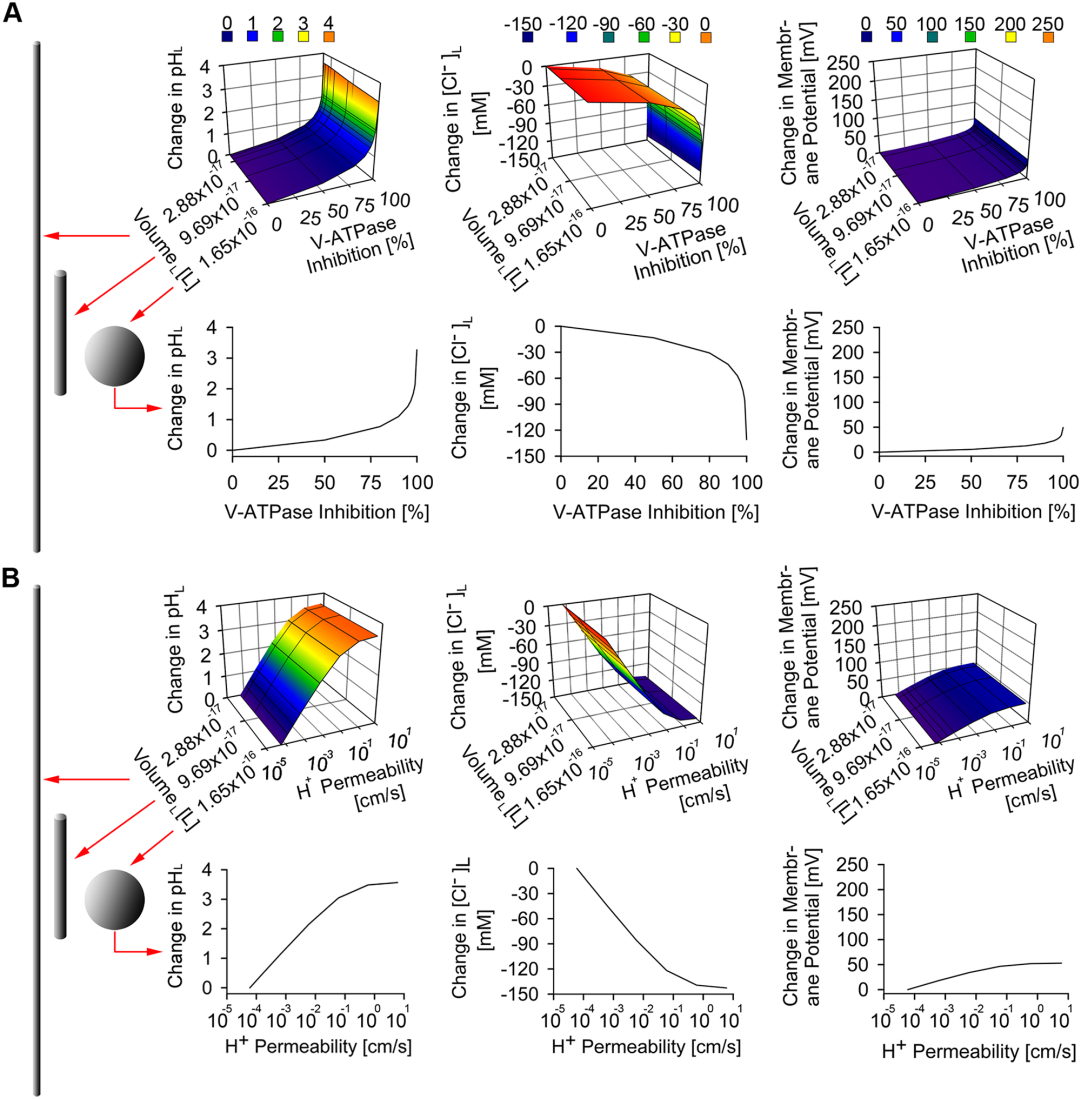
The effects of individual lysosomal ion stressors on spherical versus tubular lysosomal physiology. (A) The effect of inhibiting the number of V-ATPase molecule per lysosome showed significant changes in lysosomal pH and Cl^-^, with minimal change in membrane potential in both spherical and tubular lysosomes. (B) The effect of increasing proton-specific membrane permeability per lysosome showed significant changes in lysosomal pH and Cl^-^, with minimal change in membrane potential in both spherical and tubular lysosomes. In both (A) and (B), only a slight difference is observed in the effect of the lysosomal ion stressors on spherical versus tubular lysosomal physiology.

First, the effects of stress inducers that perturb lysosomal ion transporters (V-ATPase, CLC7, and membrane proton permeability) and ion content (cytoplasmic chloride) were studied in the context of a spherical lysosome (Fig 2A). Among the stressors we modeled, maximum reduction in the number of V-ATPase molecule per lysosome induced significant physiological perturbation in a spherical lysosome, as evidenced by the changes in lysosomal pH and Cl^-^ accumulation from their respective baseline values of 4.53 pH unit and 224.6 mM of an unperturbed lysosome (Fig 2A). We observed a similar physiological perturbation following the modeling of the effect of increasing the lysosomal membrane proton permeability on lysosomal ion homeostasis (Fig 2B). This indicates that lysosomal stressors that directly affect lysosomal proton level by perturbing either proton influx or efflux have similar effect not only on lysosomal pH, but also on lysosomal chloride homeostasis. To the contrary, CLC7 and cytoplasmic Cl^-^ stressors induced comparatively less perturbation to the overall lysosomal physiology (S1 Fig) as they only affected lysosomal pH and membrane potential when chloride transport or concentrations were completely abolished.

To understand how the same lysosomal ion stressors affect lysosomal ion homeostasis of different lysosomal morphologies, the lysosomal ion stressors were each varied along with lysosomal volume stressors that simultaneously reduced lysosomal radius and volume from an unperturbed lysosomal radius of 340 nm and a corresponding volume of 1.65x10^-16^ L, to 40 nm and 2.88x10^-17^ L, respectively. These dimensional changes capture the geometry of tubular lysosomes [57], which are narrower and more elongated than the typical, spherical lysosomes [44]. In spite of the lysosomal radius and volume differences, tubular lysosomes have the same surface area as spherical lysosomes. This assumption is reasonable because balance in cellular membrane material is maintained as a result of the regulation of membrane trafficking upon tubular lysosome mediated exocytosis of endocytosed material from the subcellular spherical lysosome to the plasma membrane. Notwithstanding, based on our results, the lysosomal ion stressors had similar effects on the physiology of tubular lysosome as they had on spherical lysosome, based on very similar changes in lysosomal pH, chloride, and membrane potential (Fig 2).

### The effects of lysosomal swelling on lysosomal ion homeostasis

**Fig 3:**
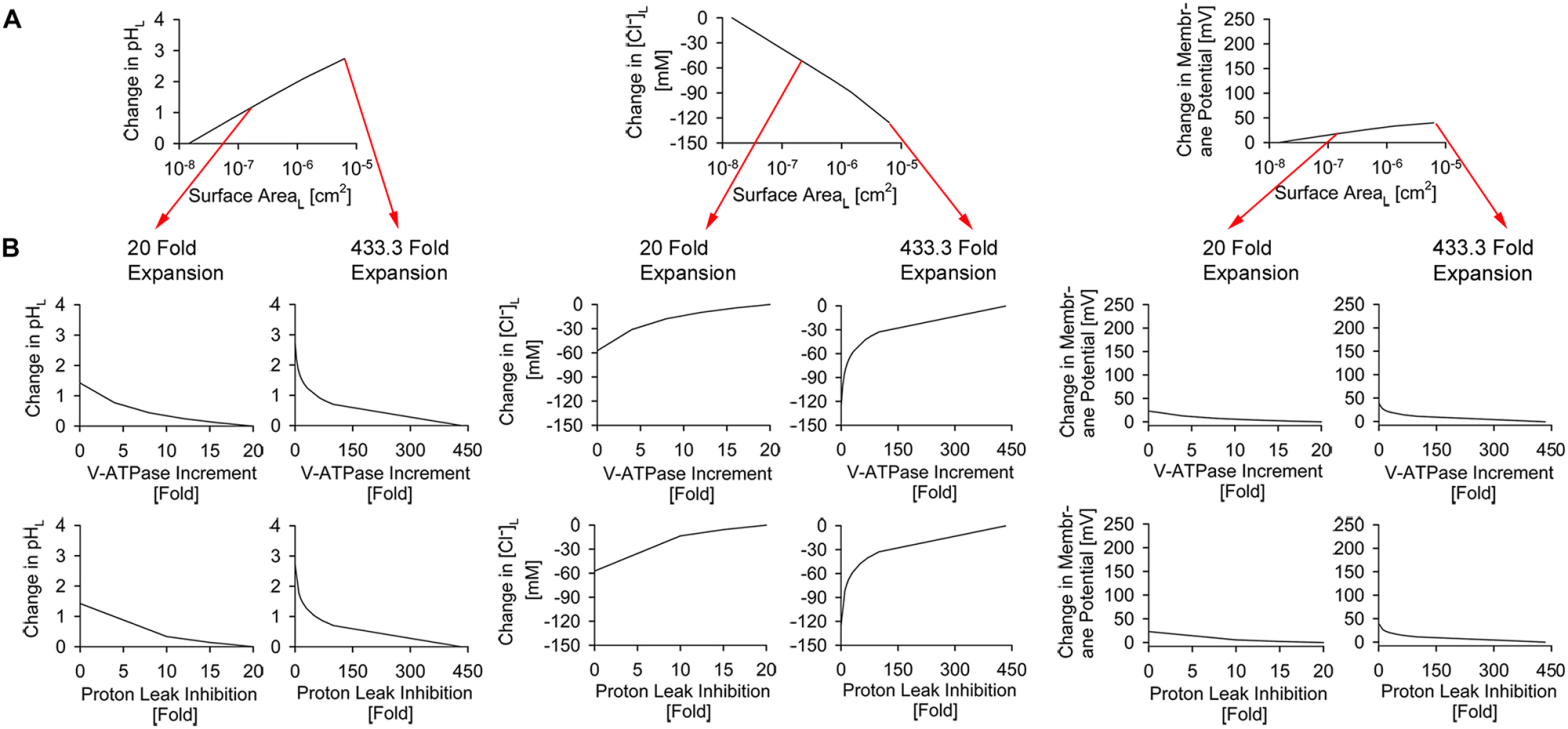
The effects of lysosomal surface expansion and associated stress tolerance on lysosomal physiology. (A) Simultaneous increment in lysosomal radius and surface area, with a constant volume of 1.65x10^-16^ L induced progressive perturbation in lysosomal pH, Cl^-^, and membrane potential. (B) Individual effects of varying V-ATPase number and membrane proton permeability on the lysosomal physiology of surface area expansion-mediated lysosomal stress. Either increasing V-ATPase number or reducing membrane proton permeability reduces transient perturbations in lysosomal pH, Cl^-^, and membrane potential following the simultaneous increment of lysosomal radius and surface area shown in (A).

Next, we modeled the effect of lysosomal swelling on lysosomal physiology by simultaneously increasing lysosomal radius and surface area while maintaining a fixed lysosomal volume of a spherical lysosomal morphology (1.65x10^-16^ L in our model, baseline lysosome). Starting from a non-perturbed spherical lysosome (radius = 340 nm, surface area = 1.45x10^-8^ cm^2^), the lysosomal radius and the corresponding surface area were expanded up to 10 μm and 6.28x10^-6^ cm^2^ (433.3 fold change), respectively. Such lysosomal swelling led to changes in lysosomal pH, Cl^-^ accumulation, and membrane potential (Fig 3A). Thus, lysosomal expansion compromised the maintenance of physiological lysosomal ion homeostasis, similar to that of V-ATPase inhibition and membrane proton permeabilization in tubular and spherical lysosomes (Fig 2).

### The mechanisms of lysosomal stress tolerance in response to lysosomal swelling

To understand the mechanism by which cells can withstand stress generated from simultaneous lysosomal radius and surface area expansion, we identified V-ATPase numbers and membrane proton permeability as key parameters that mediate lysosomal stress tolerance; which we refer to as “lysosomal stress tolerance inducers”. To illustrate this, V-ATPase number and membrane proton permeability were individually increased and decreased, respectively, 0 to 20 and 0 to 433.3 fold from their respective baseline input values. This was performed in order to maintain a constant number of proton influx and efflux mediated by V-ATPase and membrane proton permeability, respectively, for a given lysosomal surface area. We observed that physiological ion homeostasis was fully restored when either the number of V-ATPase molecules or the membrane proton permeability was kept proportional to the lysosomal surface area expansion at a fixed lysosomal volume of 1.65x10^-16^ L (Fig 3B).

### The effects of altering multiple lysosomal ion transport pathways on spherical, tubular, and disc-shaped lysosomal physiology

**Fig 4:**
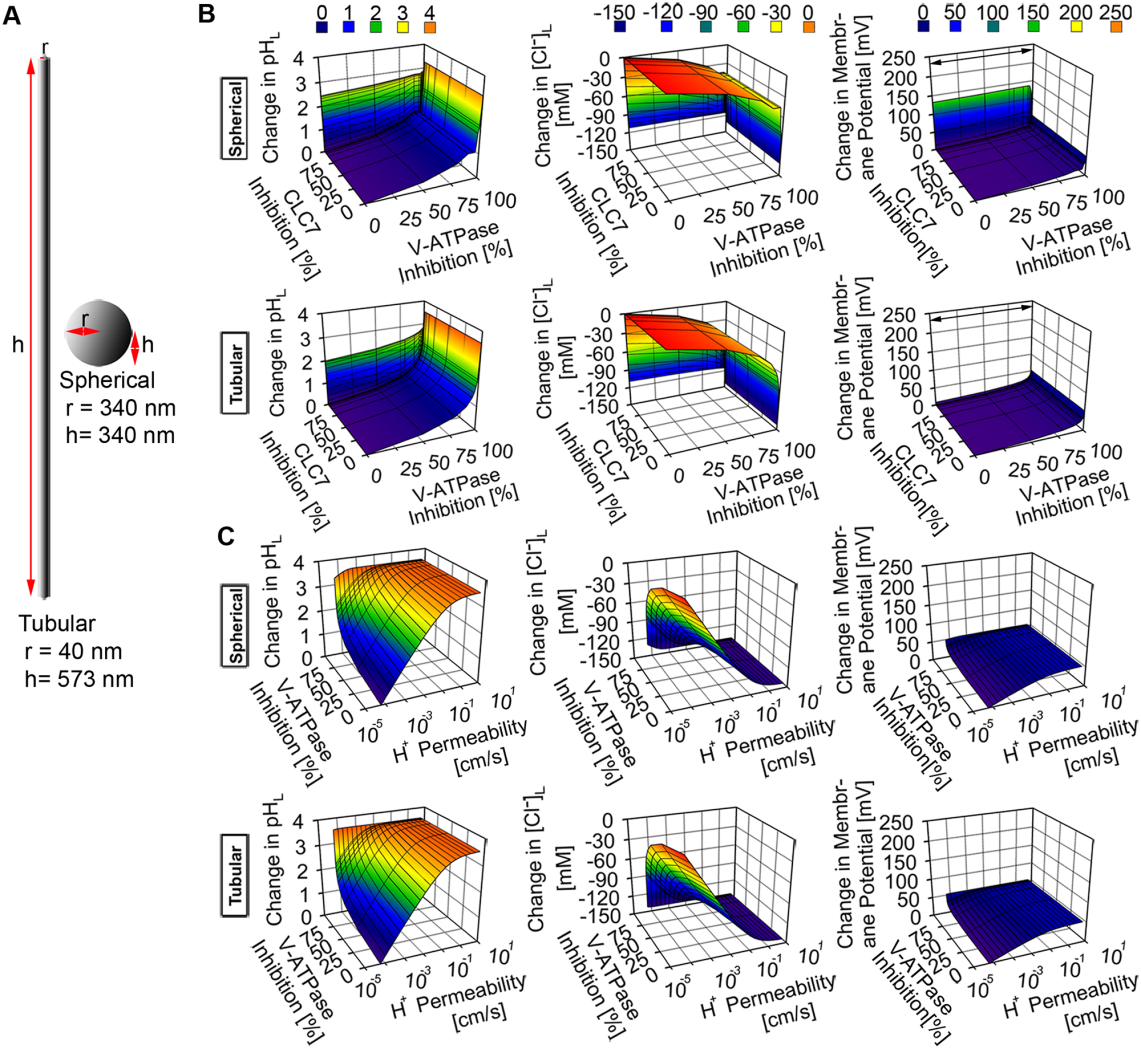
The effect of a combination of lysosomal ion stressors in spherical versus tubular lysosomes. (A) Dimensions of tubular and spherical lysosomes. (B) The simultaneous inhibitions of V-ATPase and CLC7 numbers induced changes in both spherical and tubular lysosomes with minimal difference between the two lysosomal morphologies. The increment in V-ATPase inhibition induced the most significant change while only the complete depletion of CLC7 induced physiological perturbation which mainly arose from the significant change in membrane potential (> 250 mV, as indicated by the black arrow sign). (C) The simultaneous inhibition of V-ATPase and membrane proton permeabilization induced very similar and significant changes in the overall physiology of both spherical and tubular lysosomes.

**Fig 5:**
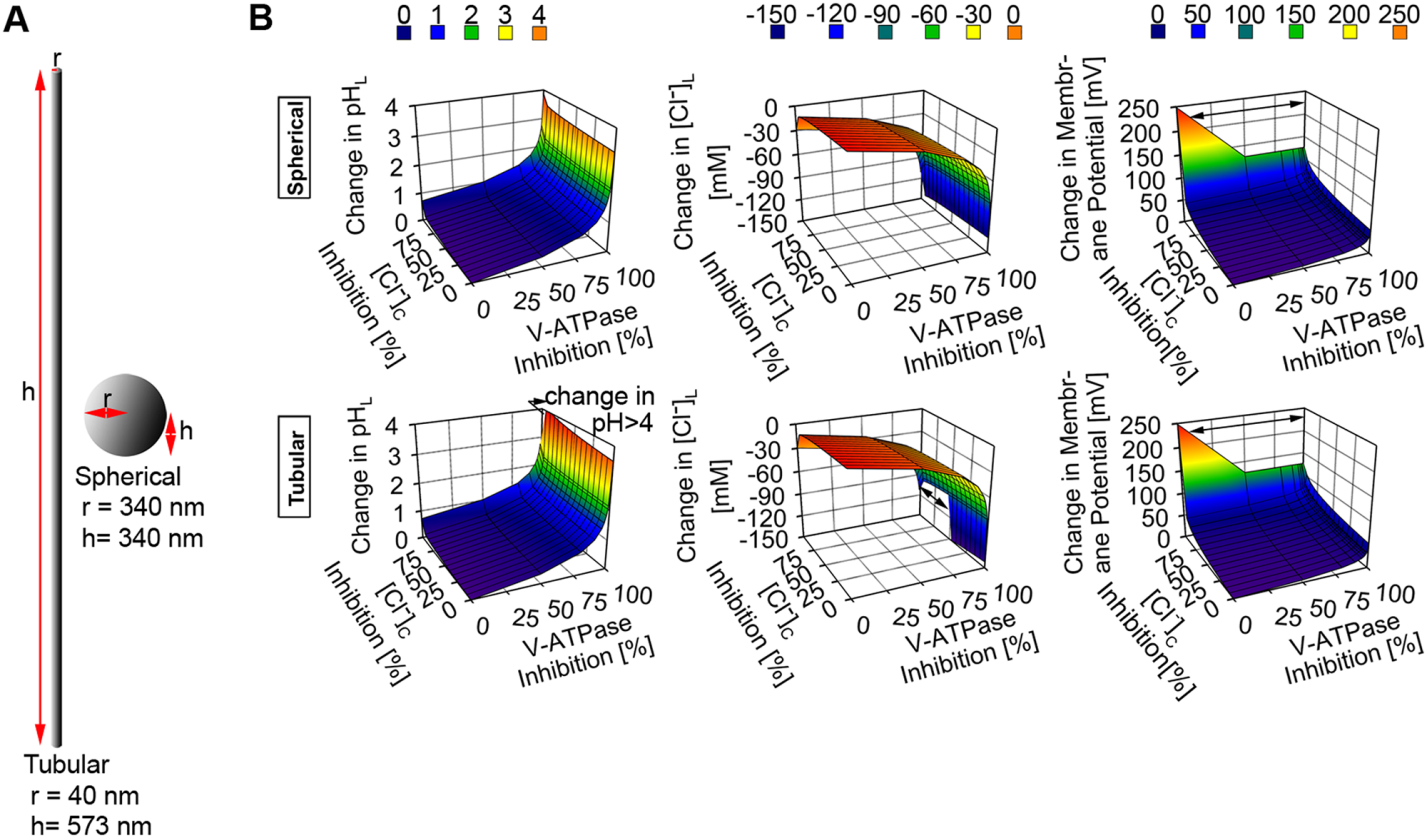
The effect of a simultaneous inhibition of the transport of chloride and proton ions in spherical versus tubular lysosomes. (A) Dimensions of tubular and spherical lysosomes. (B) The simultaneous inhibitions of the cytoplasmic chloride and V-ATPase number per lysosome induced significant changes in lysosomal pH, Cl^-^, and membrane potential. The effect was magnified in the tubular lysosome where the > 4 pH unit increment in lysosomal pH, > 150 mM reduction in lysosomal Cl^-^ accumulation, and > 250 mV increment in membrane potential were observed (as indicated by the black arrow signs).

**Fig 6:**
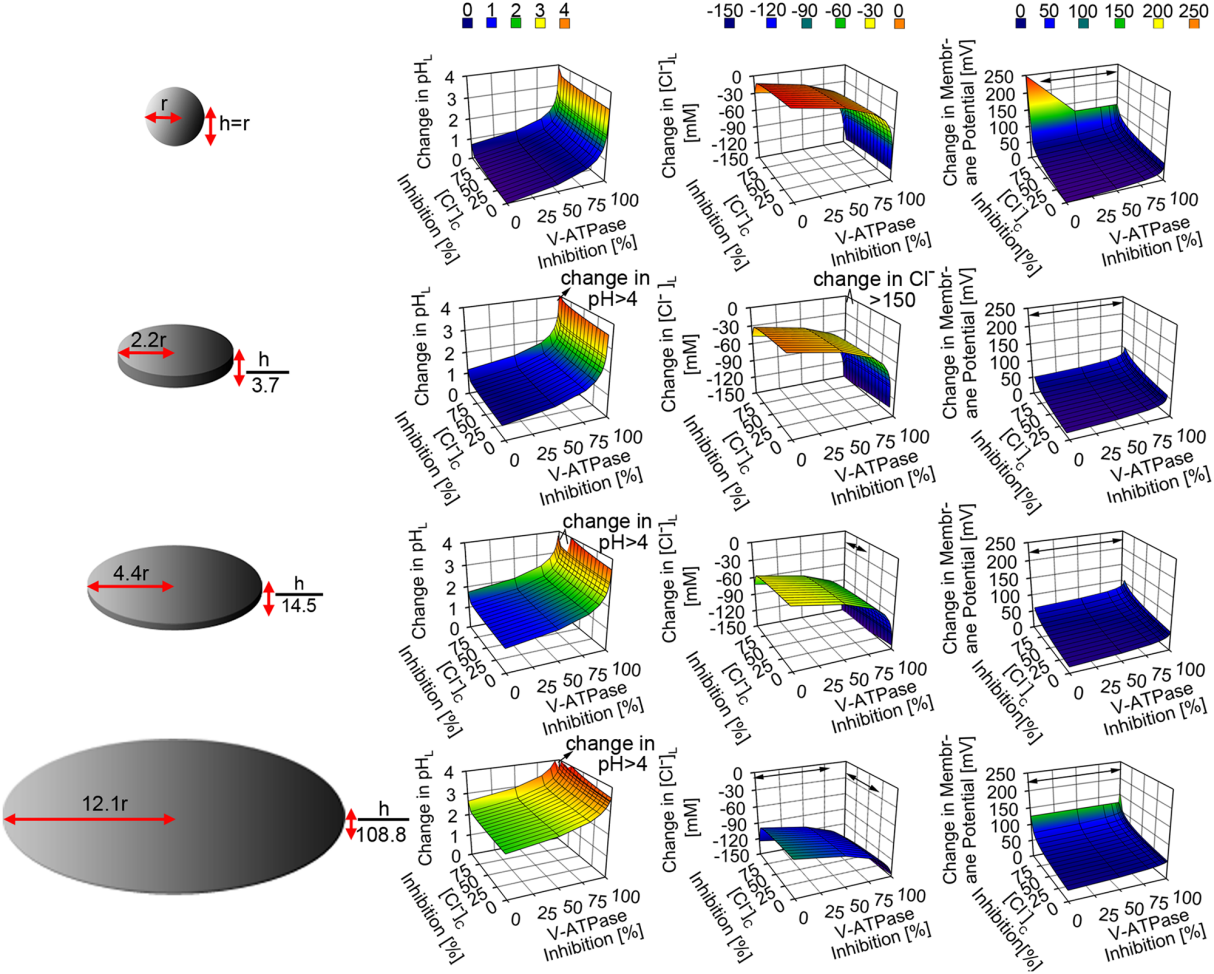
The effect of a simultaneous inhibition of proton and chloride transport in spherical versus various sized disc-shaped lysosomes. The simultaneous inhibitions of the cytoplasmic chloride and V-ATPase number per lysosome induced significant changes in lysosomal pH, Cl^-^, and membrane potential. The effect was more magnified in the disc-shaped lysosomes with lysosomal radius (> 2.2 fold) and height (< 3.7 fold) than in the spherical lysosome where the > 4 pH unit increment in lysosomal pH, > 150 mM reduction in lysosomal Cl^-^ accumulation, and > 250 mV increment in membrane potential were observed (as indicated by the black arrow signs).

Next, we investigated the mechanism by which various combinations of lysosomal ion stressors exert their effects on the physiology of lysosomes with distinct morphology. Thus, we used different combinations of various ranges of lysosomal parameters to represent V-ATPase-CLC7 stressors, V-ATPase-cytoplasmic chloride stressors, V-ATPase -membrane proton permeability stressors, and CLC7-cytoplasmic chloride stressors. These stressor combinations were simulated to study their effects on the physiology of spherical, tubular, and disc-shaped lysosomes. For all of the lysosomal morphologies, maximum alteration of either CLC7 or cytoplasmic chloride parameters in combination with alteration in either V-ATPase number or membrane proton permeability generally induced perturbation of lysosomal physiology due to the increment of membrane potential by up to > 250 mV (Figs 4B, 5 and 6).

**Fig 7:**
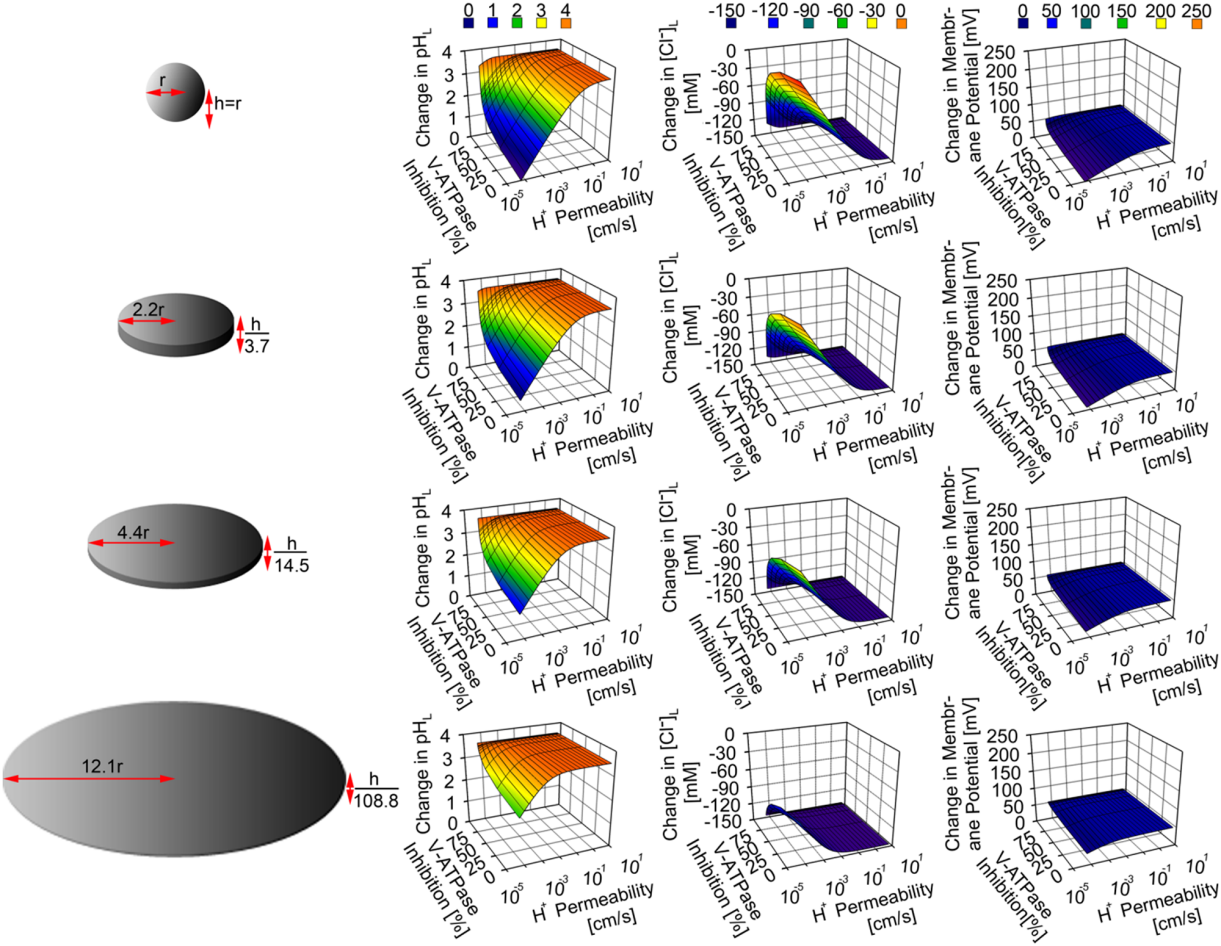
The effect of a simultaneous V-ATPase inhibition and membrane permeabilization in spherical versus various sized disc-shaped lysosomes. The alterations of the V-ATPase number and membrane proton permeability per lysosome induced very similar changes to lysosomal pH, Cl^-^, and membrane potential. For all of the disc-shaped lysosomes, as the lysosomal radius and height increased and decreased, respectively, more increment in the lysosomal pH, more reduction in the lysosomal Cl^-^ accumulation, and a slight increment in the lysosomal membrane potential were observed when there were no or very minimal alterations in both the V-ATPase number and membrane proton permeability, thus indicating the effect that the change in morphology alone has on lysosomal physiology.

Although such increment in membrane potential was associated with the increment in lysosomal pH and reduction in lysosomal chloride accumulation, greater perturbations in both variables were observed when at least either the V-ATPase number (Figs 4B, 4C, 5B, 6 and 7) or membrane proton permeability (Figs 4C and 7), (S2 and S3 Figs) were maximally altered from their respective baseline values.

Following the simultaneous reduction of both V-ATPase and CLC7 numbers, reduction of lysosomal chloride accumulation was observed to the extent of chloride efflux, in both spherical and tubular lysosomes (Fig 4B). Beyond the perturbation of lysosomal chloride level, increment in lysosomal pH was also observed. Moreover, similar perturbation of lysosomal physiology was observed when the effect of the simultaneous alteration of membrane proton permeability and CLC7 number was simulated (S2 Fig). This indicates that the presence of lysosomal ion stressors, such as V-ATPase number and membrane proton permeability stressors which directly affect proton homeostasis, alone or in combination with lysosomal ion stressors, such as cytoplasmic Cl^-^ and CLC7 number stressors, which directly affect chloride homeostasis, perturb lysosomal physiology in a very similar manner.

Regardless of lysosomal morphology, perturbations of lysosomal pH, Cl^-^ and membrane potential were generally greatest when either V-ATPase number or membrane proton permeability was simultaneously lowered or increased, respectively, along with the lowering of cytoplasmic chloride concentration from baseline values (Figs 5 and 6), and (S3 Fig). More specifically, the perturbations were greater in tubular (Fig 5) and disc-shaped lysosomes with radial expansion > 2 fold (Fig 6) than in spherical lysosome. When comparing whether V-ATPase number, membrane proton permeability, or cytoplasmic chloride level affected the aforementioned perturbation the most, it is evident that either the lowering of V-ATPase number or the increment of proton permeability similarly exerted more significant effect on lysosomal physiology (including the reduction in lysosomal chloride accumulation) than that of the lowering of cytoplasmic chloride concentration. This further corroborates the finding regarding the most significant role of net transmembrane proton flux on the regulation of lysosomal physiology.

## Discussion

Here, a physiologically-based mathematical modeling approach was used to assess the toxicological effects of drugs on lysosomes. For this purpose, we simulated the effects of altering individual or multiple parameters controlling lysosomal morphology and ion regulation on the recovery of lysosomes from a transient perturbation. Related to this, different cell types are able to tolerate lysosomal stress to different extents. While lysosomes of different cell types are known to vary in size, shape, and the molecular transport phenomena responsible for maintaining lysosomal pH, membrane potential, and ion concentrations, computational modeling approach enables the probing of the effect of variations of specific parameters on lysosomal physiology in a manner that is difficult—if not impossible—to control with pharmacological experiments.

### Simulation of cell-type dependent differences in lysosomal stress response

Variations in lysosomal morphology [62] and lysosomal protein expression [21] are manifested among different cell types. Our model simulations probed the mechanism by which such variations affected the cells’ ability to maintain lysosomal physiology. In terms of lysosomal morphology, our findings indicate that cell types that either have tubular or spherical lysosomes have very similar physiological profile characterized by the observed similar changes in lysosomal pH, Cl^-^, and membrane potential values following the introduction of various lysosomal ion stressors which induced the following lysosomal stresses: V-ATPase inhibition, CLC7 inhibition, cytoplasmic chloride inhibition, and membrane proton permeabilization. In spite of the difference in lysosomal volume between spherical and tubular lysosomal morphologies, as long as the lysosomal surface area is the same in both, their lysosomal physiology will only be dictated by the amount of active lysosomal V-ATPase and CLC7 proteins, and ion content, including cytoplasmic and lysosomal chloride and protons. Among the parameters that vary in a cell-typedependent manner, changes in the V-ATPase number and membrane proton permeability showed the most effect on lysosomal physiology as reflected by the significant changes in lysosomal pH, chloride, and membrane potential from their respective physiological baseline values. In contrast, alterations in CLC7 number and cytoplasmic chloride concentration had comparatively minimal effects on lysosomal physiology. This implies that the regulation of net proton transportation to and from the lysosome, for a given lysosomal surface area, plays a more significant role than that of net chloride transportation in the maintenance of lysosomal physiology.

Upon investigating the effect of simultaneously changing multiple, cell-type dependent lysosomal parameters, simulation results revealed that the degree of lysosomal physiology perturbation was mainly determined by changes in the V-ATPase number or membrane proton permeability. Moreover, varying either of these two parameters along with the level of cytoplasmic chloride resulted in distinct, lysosomal morphology-dependent perturbations of lysosomal pH, Cl^-^, and membrane potential as the perturbation was heightened in tubular and disc shaped lysosomes. Tubular lysosomes are prevalent in cells with less lysosomal contents [44], whereas disc-shaped lysosomes are prevalent in cells with greater lysosomal content than that of spherical lysosomes [63–65]. Thus, our findings imply that cell types with tubular or disc-shaped lysosomes may be more prone to toxic effects of lysosomotropic agents, in comparison to cell types with spherical lysosomes.

### Simulation of the effects of drugs on lysosomal physiology

Pharmacologically, various drugs can be used to disrupt lysosomal ion homeostasis, such as V-ATPase inhibitor, chloride channel inhibitor, membrane proton permeabilizer, and cytoplasmic chloride reducer [29, 40, 41, 48, 66, 67]. Moreover, there are also drugs that induce changes to lysosomal morphology by either affecting lysosomal volume or surface area [39, 43, 44]. Thus, by modeling the effects of altering lysosomal parameters, we have obtained insights into the mechanism by which lysosomal stress inducing drugs affect the regulation of lysosomal pH, Cl^-^, and membrane potential. Moreover, by altering various combinations of the lysosomal parameters, it is possible to obtain insights into the toxicological effects of drugs that exert pleiotropic effects on organelle ion homeostasis. Again, our results indicate that drugs that affect net proton flux across the lysosomal membrane, either by inhibiting the V-ATPase or by permeabilizing the lysosomal membrane to protons, exert the most significant perturbation to lysosomal physiology.

Of noteworthy significance, many lysosomotropic and cationic amphiphilic drugs [39] induce lysosomal volume expansion, which can profoundly alter lysosomal physiology in the absence of compensatory changes in either V-ATPase number or membrane proton permeability. In contrast to drugs that induce lysosomal vacuolation, drugs that reduce lysosomal volume do not exert significant physiological perturbations, as reflected by the minimum changes in lysosomal pH, Cl^-^, and membrane potential. This emphasizes the necessity of regulation of net lysosomal proton flux dictated by the number of V-ATPase, membrane proton permeability, and lysosomal surface area.

In addition, our mathematical model predictions can be tailored to investigate the mechanism by which cell-death inducing lysosomal membrane permeabilizers (LMP) affect lysosomal physiology based on various factors, such as size of LMP agent, cell-type dependent lysosomal size, and lysosomal ion content [68]. In the case where perturbed membrane proton permeability due to cholesterol imbalance is the causative agent for LMP-mediated cell death, the role of V-ATPase upregulation in lysosomal stress tolerance could be experimentally studied [69]. Alternatively, the role of cholesterol-mediated changes in the proton permeability of membranes could also be investigated in cells expressing varying V-ATPase levels.

### Relationship between lysosomal morphology and stress response

In order to understand the mechanism by which lysosomal stress response is associated with lysosomal morphology [64], we altered various combinations of lysosomal ion parameters together with geometric parameters that determine lysosomal shape. Only under very specific conditions was there a distinctively different perturbation in the physiology of tubular lysosome versus spherical lysosome (Fig 5). The perturbation of lysosomal physiology following the simultaneous presence of a membrane proton permeabilizer and a cytoplasmic chloride stressor can be associated with the cytotoxic phenomenon of LMP-mediated cytosolic acidification along with lysosomal alkalinization [68, 70–72]. This mechanism may be relevant to the selective toxicity of lysosomotropic agents to cancer cells [73], because tubular lysosomes play a significant role in the shuttling of V-ATPase to the plasma membrane, which influences the survival and proliferation of cancer cells [40, 66, 74]. Moreover, this finding could shed light on the mechanism by which certain cationic amphiphilic drugs which destabilize lysosomal membrane induce anticancer effects [75].

### Insights into how exocytosis, endocytosis, and cholesterol may affect lysosomal stress response

Based on our findings, even though exocytosis and endocytosis can affect changes in lysosomal volume and surface area, significant effects on lysosomal pH, membrane potential, and chloride regulation are only observed in the context of specific number of V-ATPases, membrane proton permeability, and cytoplasmic chloride level per lysosome. More specifically, the simultaneous inhibitions in V-ATPase number and cytoplasmic chloride resulted in high increment in lysosomal pH, reduction in lysosomal chloride accumulation, and increment in membrane potential (Figs 5 and 6). Endocytosis or exocytosis affect lysosomal morphology through changes in membrane surface area, and hence could be linked to specific toxicological manifestations of lysosomotropic drugs (Fig 6). The effect of endocytosis or exocytosis on lysosomal stress can be offset, either through V-ATPase upregulation or reduction in membrane proton permeability. Regulation of membrane proton permeability can be induced by cholesterol regulation. Because cholesterol transportation to and from the lysosomes is facilitated by Niemann-Pick type C1 (NPC1) proteins [76, 77], these proteins themselves could also contribute to lysosomal stress response [78] during endocytosis or exocytosis.

### Insights into the TFEB lysosomal stress response mechanism

Our finding suggests that V-ATPase expression plays a critical role in restoring lysosomal ion homeostasis following a transient perturbation. Transcription factor EB (TFEB) is a major transcriptional regulator of V-ATPase expression [79, 80]. Thus, the upregulation of V-ATPase may be sufficient to explain how TFEB upregulation may confer resistance to perturbations of lysosomal physiology. Although previous findings have indicated that ions or channels involved in cation transport may explain how TFEB induces its role [79, 80], appropriate interpretation of the experiments would depend on knowing any effects on the function of the V-ATPase. This is especially important as there have been reports [43, 81, 82] showing TFEB mediated upregulation of lysosomal genes as a stress tolerance mechanism following the accumulation of weakly basic drugs. However, the induced stress tolerance may not necessarily be sufficient enough to restore full lysosomal function [81]. Thus, the extent of TFEB nuclear translocation and its transcriptional activity following lysosomal stress must be well understood in relation to the upregulation of V-ATPase expression. Indeed, additional cell growth regulators, such as mammalian target of rapamaycin (mTOR) complex 1 (mTORC1), which is associated with TFEB and V-ATPase regulation [34], could be investigated to quantify the relationship between TFEB nuclear translocation, V-ATPase expression, and amount and type of endocytosed or internalized lysosomal stress inducer.

## Conclusion

To conclude, a mathematical model was used to probe the mechanistic determinants of drug induced lysosomal stress and stress resistance, in the presence of individual as well as combination of lysosomal stressors. By testing the effects of different parameters that determine organelle morphology and ion transport, the net proton flux with respect to lysosomal surface area emerged as the key parameter affecting lysosomal stress sensitivity and resistance. Accordingly, the expression levels of V-ATPase can act in concert with the regulation of membrane proton permeability to determine the differential sensitivity of different cell types to the toxic effects of lysosomotropic drugs.

## Supporting information

S1 Fig The effects of individual lysosomal chloride transportation stressors on the physiology of spherical versus tubular lysosomes. (A) Modeling the effect of varying CLC7 number on lysosomal pH, Cl^-^, and membrane potential with respect to different lysosomal morphology. Maximum depletion of CLC7 has a slightly more pronounced effect on spherical lysosomal physiology than on tubular lysosomal physiology, as reflected by the maximum increment in membrane potential (> 250 mV, represented by the black arrow). (B) Modeling the effect of varying cytoplasmic chloride concentration on lysosomal pH, Cl^-^, and membrane potential with respect to different lysosomal morphology. Maximum depletion of cytoplasmic chloride induced maximum increment in membrane potential (up to 248.3 mV), with minimal changes in lysosomal pH and Cl^-^ accumulation.
(TIF)

S2 Fig The effect of a simultaneous CLC7 inhibition and membrane proton permeabilization on the physiology of spherical versus tubular lysosomes. (A) Lysosomal dimensions used to generate spherical and tubular lysosomes. (B) Modeling the effect of simultaneous variations of CLC7 number and membrane proton permeability on lysosomal pH, Cl^-^, and membrane potential. The simultaneous maximum depletion of CLC7 number and increment in membrane proton permeability (> 6x10^-3^ cm/s in the case of the effect on lysosomal pH and Cl^-^, and > 6 x10^-5^ cm/s in the case of the effect on lysosomal membrane potential) induces > 4 pH unit increment in lysosomal pH, > 150 mM reduction in lysosomal Cl^-^ accumulation, and > 250 mV increment in membrane potential, as indicated by the black arrows. Although the overall effect of these stressors is very similar in both spherical and tubular lysosomal physiology, the effect is slightly more pronounced on spherical lysosomal physiology.
(TIF)

S3 Fig The effect of a simultaneous cytoplasmic chloride inhibition and membrane proton permeabilization on spherical versus disc-shaped lysosomal physiology. Cytoplasmic chloride concentration and membrane proton permeability were simultaneously varied in spherical and different sized disc-shaped lysosomes to observe their combined effects on lysosomal pH, Cl^-^, and membrane potential. Simultaneously increasing the cytoplasmic chloride inhibition (> 80%) and membrane proton permeability (> 0.06 cm/s) induced > 4 pH unit increment in lysosomal pH and > 150 mM reduction in lysosomal Cl^-^ accumulation, represented by the black arrows. For all lysosomal morphologies, maximum increment in membrane potential (> 250 mV, represented by the black arrows) is observed at maximum cytoplasmic chloride inhibition. However, the perturbation in lysosomal physiology was generally pronounced as the lysosomal radius and surface area expansions were increased.
(TIF)

S1 Text Lysosomal ion transport model description. (DOCX)
