# Supplementary figures and images for: The physiological determinants of drug-induced lysosomal stress resistance
Source: PLoS One. 2017 Nov 8;12(11):e0187627. doi: 10.1371/journal.pone.0187627 (PMC5678708; doi:10.1371/journal.pone.0187627)

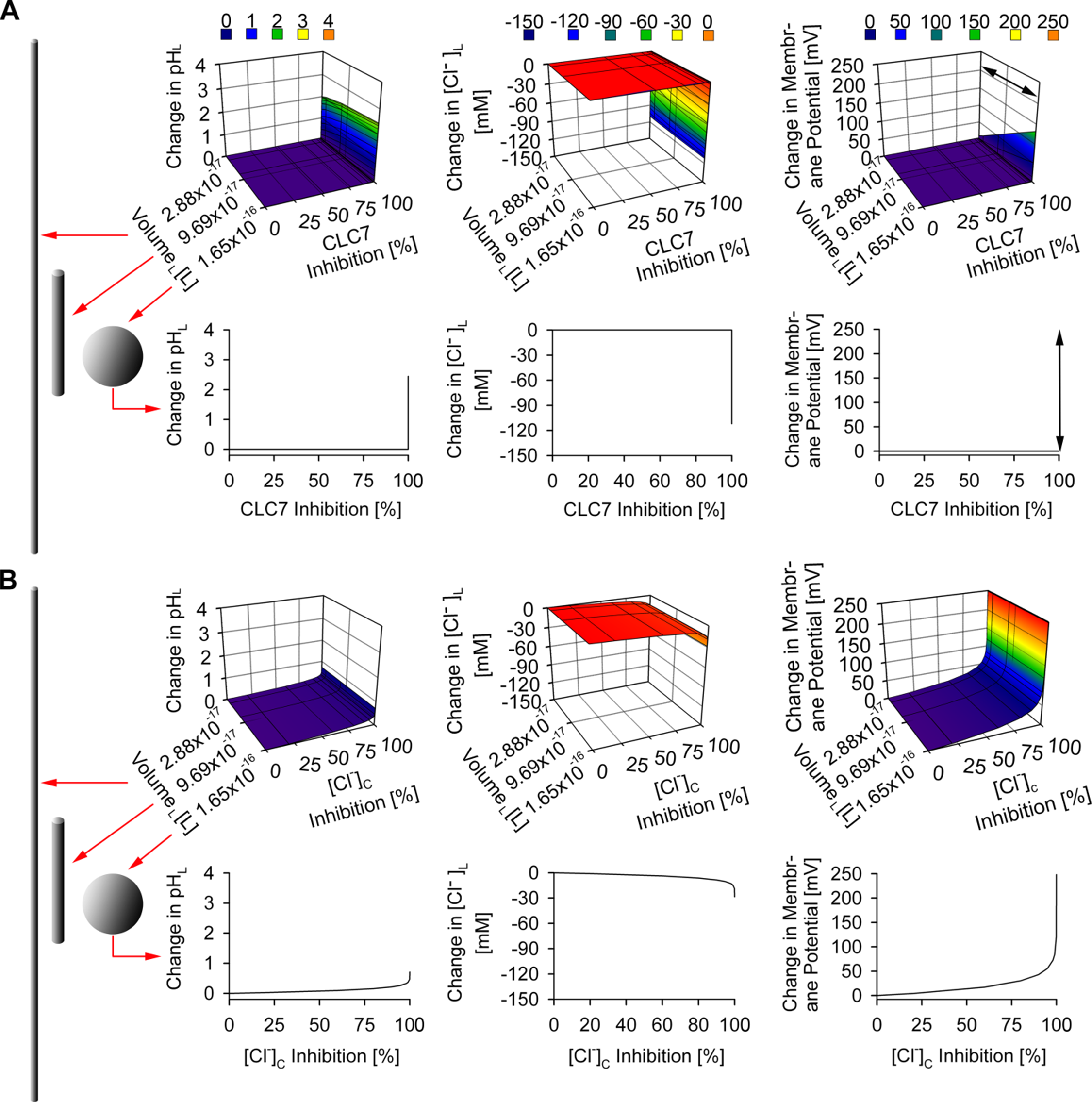

Supplement: S1 Fig — (A) Modeling the effect of varying CLC7 number on lysosomal pH, Cl-, and membrane potential with respect to different lysosomal morphology. Maximum depletion of CLC7 has a slightly more pronounced effect on spherical lysosomal physiology than on tubular lysosomal physiology, as reflected by the maximum increment in membrane potential (> 250 mV, represented by the black arrow). (B) Modeling the effect of varying cytoplasmic chloride concentration on lysosomal pH, Cl-, and membrane potential with respect to different lysosomal morphology. Maximum depletion of cytoplasmic chloride induced maximum increment in membrane potential (up to 248.3 mV), with minimal changes in lysosomal pH and Cl- accumulation. (TIF) [file pone.0187627.s001.tif]

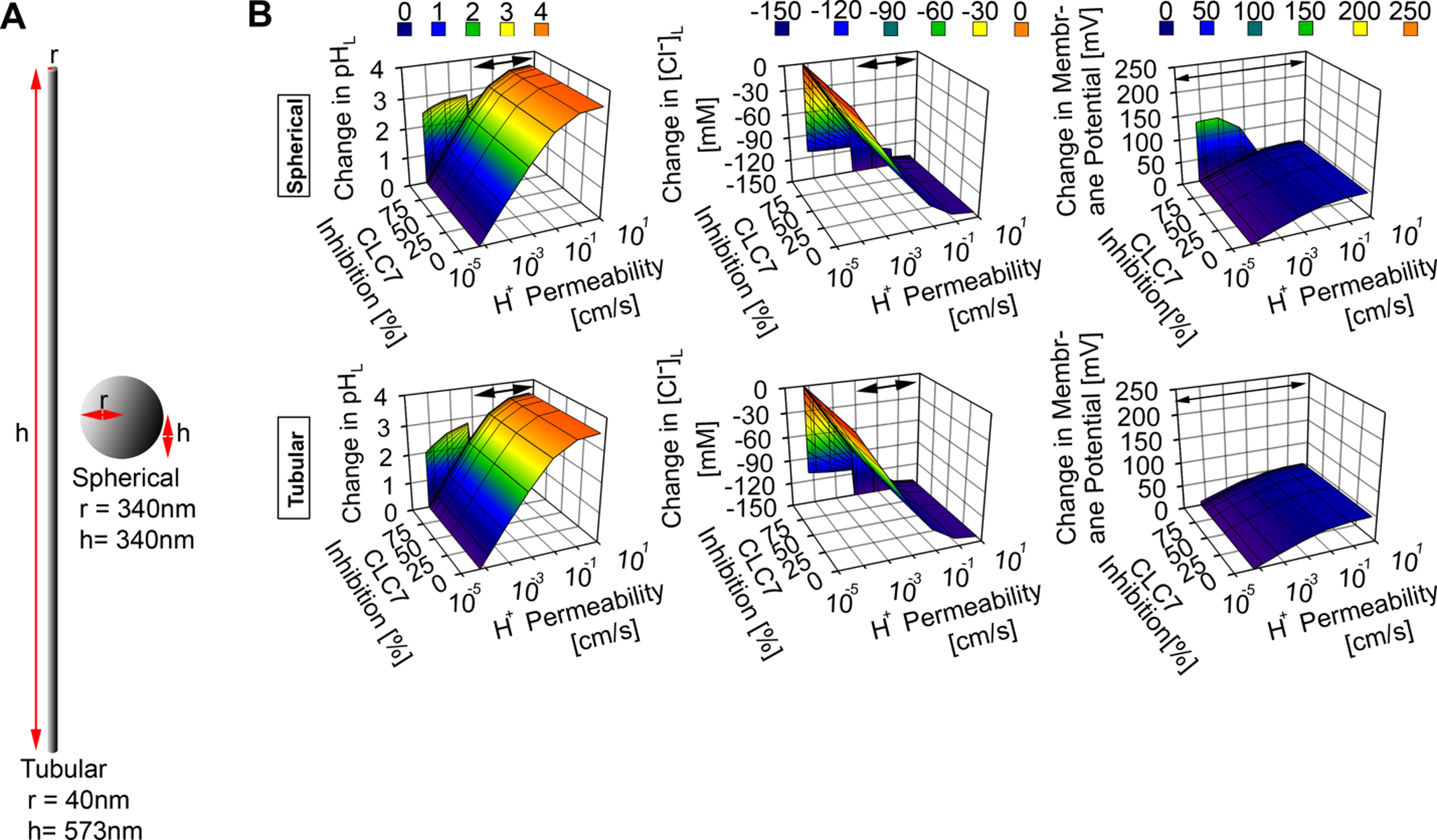

Supplement: S2 Fig — (A) Lysosomal dimensions used to generate spherical and tubular lysosomes. (B) Modeling the effect of simultaneous variations of CLC7 number and membrane proton permeability on lysosomal pH, Cl-, and membrane potential. The simultaneous maximum depletion of CLC7 number and increment in membrane proton permeability (> 6x10-3 cm/s in the case of the effect on lysosomal pH and Cl-, and > 6 x10-5 cm/s in the case of the effect on lysosomal membrane potential) induces > 4 pH unit increment in lysosomal pH, > 150 mM reduction in lysosomal Cl- accumulation, and > 250 mV increment in membrane potential, as indicated by the black arrows. Although the overall effect of these stressors is very similar in both spherical and tubular lysosomal physiology, the effect is slightly more pronounced on spherical lysosomal physiology. (TIF) [file pone.0187627.s002.tif]

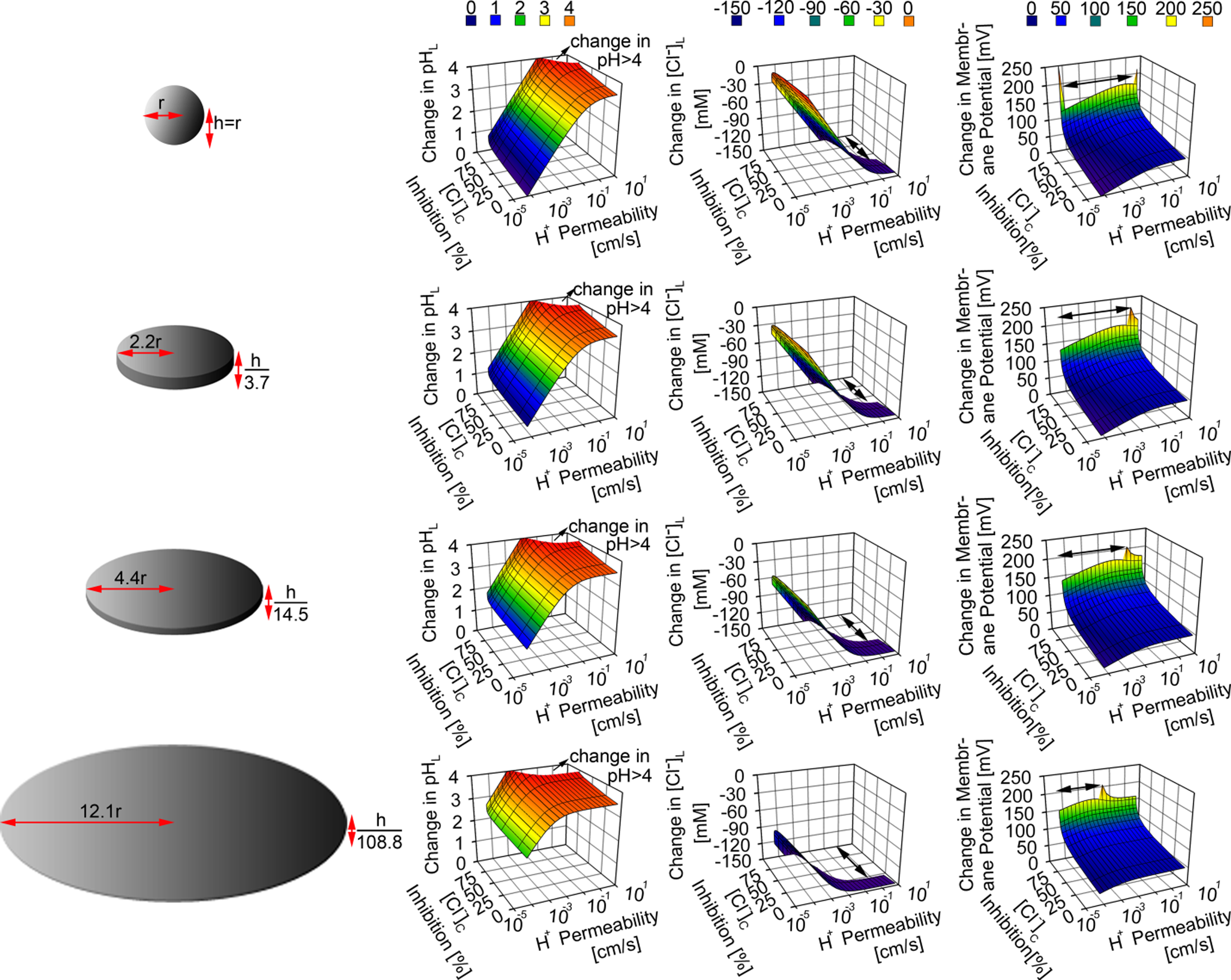

Supplement: S3 Fig — Cytoplasmic chloride concentration and membrane proton permeability were simultaneously varied in spherical and different sized disc-shaped lysosomes to observe their combined effects on lysosomal pH, Cl-, and membrane potential. Simultaneously increasing the cytoplasmic chloride inhibition (> 80%) and membrane proton permeability (> 0.06 cm/s) induced > 4 pH unit increment in lysosomal pH and > 150 mM reduction in lysosomal Cl- accumulation, represented by the black arrows. For all lysosomal morphologies, maximum increment in membrane potential (> 250 mV, represented by the black arrows) is observed at maximum cytoplasmic chloride inhibition. However, the perturbation in lysosomal physiology was generally pronounced as the lysosomal radius and surface area expansions were increased. (TIF) [file pone.0187627.s003.tif]
