# Supplementary material for: The physiological determinants of drug-induced lysosomal stress resistance
Source: PLoS One. 2017 Nov 8;12(11):e0187627. doi: 10.1371/journal.pone.0187627 (PMC5678708; doi:10.1371/journal.pone.0187627)
Supplement: S1 Text — (DOCX) [file pone.0187627.s004.docx]

**Supporting information**

**The physiological determinants of drug-induced lysosomal stress resistance**

Tehetina Woldemichael^1^ and Gus R. Rosania^2*^

^1^Biophysics Program, College of Literature, Science, and the Arts, University of Michigan, Ann Arbor, Michigan, United States of America

^2^Department of Pharmaceutical Sciences, College of Pharmacy, University of Michigan, Ann Arbor, Michigan, United States of America

* Corresponding author:

E-mail: [grosania@umich.edu](mailto:grosania@umich.edu) (GRR)

**Lysosomal ion transport model description**

The transportation of ions to and from the lysosome induces charge, ΔQ, and membrane potential difference, ΔΨ, between the lysosome and the cytoplasm. By definition, membrane potential difference, which simply is referred hereon as membrane potential, across a given membrane is the difference between the potential of one side of the membrane and that of the other side of the membrane (i.e. the lysosome and the cytoplasm in our case). It can be calculated using the following relationship between the charge of a given ion(s) and capacitance, assuming that the lipid bilayer of lysosomal membrane can serve as a parallel plate capacitor:

$\Delta\Psi= \frac{\Delta Q}{C^{'}}$ (1)

Where C’ is the specific bilayer capacitance of the lysosomal membrane per unit area of the lysosomal surface area, which represents the measure of the net charge transported, ΔQ, in units of Coulomb, from one side of the membrane to the other resulting in membrane potential, ΔΨ, across the membrane, in units of mV. For biological membranes, C’ has been experimentally approximated to 1μF/cm^2^ [1, 2].

Charge on a mole of a monovalent ion is represented by Faraday’s constant, F, which equals 96485 Coulomb/mol. Moreover, depending on the charge and valence, Z_i_, of the monovalent ion “i", F will be + or -; where the earlier is for a cation and the latter for an anion. Thus, using F we can obtain the charge of the ions in the cytoplasm and the lysosome from the cytoplasmic and lysosomal ion concentration, respectively, in units of Coulomb.

$\Delta Q=F\times( \sum_{i} Z_{i}\times[i]_{C}{\times V}_{C}-\sum_{i} Z_{i}\times\left[ i]_{L}{\times V}_{L} \right)$ (2)

With [i_C_] and [i_L_] being the net concentration in the cytoplasm and lysosome in units of Molar, respectively. Both terms are multiplied by their respective compartmental volume to convert the units of Molar to mol. In both compartments, the net concentration comprises of permeable and impermeable ions.

Furthermore, we utilize a physiological sign convention that the membrane potential is more positive or negative if there is more cation or anion, respectively, in the internal compartment (i.e. lysosome, in our case), than in the external compartment (i.e. cytoplasm, in our case). Thus, equation 2 is re-written as:

$\Delta Q=F\times(\sum_{i} Z_{i}\times[i]_{L}\times V_{L}-\sum_{i} Z_{i}\times\left[ i]_{C}{\times V}_{C} \right)$ (3)

More specifically, the following relationship is obtained to define the ion content in the cytoplasmic compartment: $\sum_{i} [i]_{C}{\times V}_{C}=\sum_{i} ([i]_{C}{\times V}_{C})_{f}- \sum_{i} ([i]_{C}\times V_{C})_{o}$ (4)

Where the subscript f denotes the final value of the permeable ions in units of mole, and the subscript o denotes the initial value of the impermeable ions in units of mole. However, because the cytoplasmic volume is comparatively big, we assume that the concentration of cytoplasmic ions remains more or less constant. Thus, we set: $\sum_{i} ([i]_{C}{\times V}_{C})_{o}=0$

Similar to equation 4, we have the following relationship for the case of lysosomal ion content: $\sum_{i} [i]_{L}{\times V}_{L}=\sum_{i} ([i]_{L}\times V_{L})_{f}-\sum_{i} ([i]_{L}{\times V}_{L})_{o}$ (5)

Thus, by substituting the terms in equation 3 with those in equations 4 and 5, we obtain; $\Delta Q=F\times(\sum_{i} Z_{i}\times([i]_{L}\times V_{L})_{f}-\sum_{i} Z_{i}\times(\left[ i]_{L}{\times V}_{L})_{o} \right)$ (6)

In addition, because we treat the lysosomal volume as a parameter, equation 6 can be re-written as: $\Delta Q=F{\times V}_{L}\times((\sum_{i} Z_{i}\times[i]_{L})_{f}-\sum_{i} Z_{i}\times(\left[ i]_{L})_{o} \right)$ (7)

Moreover, for the purpose of distinguishing notations, $\sum_{i} Z_{i}\times(\left[ i]_{L})_{o} \right)$ is replaced by “B”. This term is known as Donnan particles, which is represented in units of Molar. Thus, equation 7 is re-written as: $\Delta Q=F{\times V}_{L}\times((\sum_{i} Z_{i}\times[i]_{L})_{f}-B)$ (8)

Thus, by plugging equation 8 into equation 1, membrane potential is equated as;

$\Delta\Psi= \frac{F{\times V}_{L}}{C^{'}}\times((\sum_{i} Z_{i}\times[i]_{L})_{f}-B)$ (9)

As previously mentioned, because the capacitance is a measurement per unit area of the lysosomal surface, equation 9 must be multiplied by the total lysosomal surface area (S), in units of cm^2^, to obtain total capacitance per lysosome, hence the total membrane potential.

$\Delta\Psi= \frac{F\times V_{L}}{C^{'}\times S}*((\sum_{i} Z_{i}\times[i]_{L})_{f}-B)$ (10)

Furthermore, the final luminal ion concentrations [i_L_]_f_ can be classified into cations and anions:

$\Delta\Psi= \frac{F\times V_{L}}{C^{'}\times S}*\left[ \left( \sum_{i} Z_{i}\times[cations]_{i}+\sum_{i} Z_{i}\times[anions]_{i} \right)-B \right]$ (11)

In addition, the Donnan particles (B), in units of Molar, are explicitly defined using the initial lysosomal contents, including the net change in the intrinsic surface potential:

$B= [H^{+}]_{L,initial}+ [K^{+}]_{L,initial}+ [\mathrm{Na}^{+}]_{L,initial}- [Cl^{-}]_{L,initial}-\frac{C^{'}\times S}{F{\times V}_{L}}\times\{\left( \Psi_{\mathrm{in}}-\Psi_{\mathrm{out}} \right)+\Psi_{\mathrm{initial}}\}$ (12)

With the subscript (initial) denoting the fixed initial luminal content, (Ψ_in_) and (Ψ_out_) being the intrinsic surface potentials for inner and outer leaflets in units of mV, respectively, of the lysosomal membrane, as estimated in the literature [3], (Ψ_initial_) being the initial membrane potential, which is set to 0 mV for the purpose of maintaining initial electroneutrality.

Substituting equation 12 into equation 11 will give:

$\Delta\Psi= \frac{F{\times V}_{L}}{C^{'}S}\times\{[H^{+}]_{L,final}-[Cl^{-}]_{L,final}- \left[ H^{+}]_{L,initial}+ \left[ Cl^{-}]_{L,initial} \right\}+ \Psi_{\mathrm{in}}-\Psi_{\mathrm{out}} \right\}$ (13)

With the subscript (final) denoting the luminal contents at a given time “t”.

**Modeling ion transportation.** Briefly, in this model, V-ATPase is an electrogenic proton pump which lowers the lysosomal pH by working against a proton motive force build-up that arises from membrane potential and proton concentration gradient. To capture this phenomenon, the rate of proton influx, J_HVATP_, by a single V-ATPase molecule per lysosome per second was incorporated to the model based on the instantaneous transmembrane pH gradient, ΔpH, and membrane potential, ΔΨ, (equation 14). This rate was multiplied by the total number of active V-ATPase molecules in a lysosome, N_VATP_ [3, 4] to obtain the total number of protons inserted into the lysosome in units of molecules per second (H_pump_), (equation 14).

$H_{\mathrm{pump}}=N_{\mathrm{VATP}}\times J_{H_{\mathrm{VATP}}}\left( \Delta pH, \Delta\Psi\right)$ (14)

Following proton pump by the V-ATPase, membrane potential would increase in the absence of some other mechanism that dissipates the increment. Physiologically, membrane potential is dissipated by the efflux of cations from the lysosomes, or the influx of anions. In this regard, CLC7 plays a dominant role [4]. Accordingly, the model represents the rate of proton removal per second, J_Cl,H_CLC7_, as well-elaborated in a previously published model [3], by a single CLC7 molecule per lysosome as a function of chloride concentration gradient dictated by cytoplasmic and lysosomal chloride concentrations, Cl_C_ and Cl_L_, respectively, and electrical gradient dictated by ΔΨ, (equation 15). This rate was multiplied by the total number of CLC7 molecules per lysosome, N_ClC7_, [3] in order to obtain the total amount of protons removed in units of molecules per second by CLC7, H_ClC7_,(equation 15).

$H_{ClC7}= N_{ClC7}\times J_{Cl, H_{ClC7}}\left( \Delta pH, \mathrm{Cl}_{L} , \mathrm{Cl}_{C}, \Delta\Psi\right)$ (15)

In addition to CLC7, membrane potential dissipation is also facilitated by the process of proton transportation from the lysosome to the cytoplasm. In order to capture the passive diffusion of protons across the semi-permeable lysosomal membrane, the Goldman-Hodgkin-Katz (GHK) flux equation [5] was used. GHK flux equation was derived to model the flux of an ion “i”, j_i_, dictated by chemical, dC/dX, and electrical potential, dΨ/dX, gradients across a biological membrane:

$j_{i}= -Z_{i}{\times u}_{i}\times R\times T\times\left( \frac{\mathrm{dC}}{\mathrm{dX}}+ Z_{i}\times C\times\frac{F}{R\times T}\times\frac{d\Psi}{\mathrm{dX}} \right)$ (16)

Where Z is charge and valence of the ion, “i”, C is the ion concentration, R is gas constant, T is absolute temperature, i.e. 0 Kelvin, and u_i_ is the electro-kinetic ion mobility and is further related to the diffusion coefficient, D_i_, of the ion by the following Einstein relation:

$u_{i}= D_{i}\times\frac{Z_{i}\times F}{R\times T}$ (17)

Assuming a constant electric field across a homogenous membrane with thickness of l, the above equation is integrated at steady state to give the flux of ion “i" across the membrane using boundary conditions where at x = 0, the ion concentration = C_0_ (i.e. ion concentration before transportation at one side of the membrane) and at x = l, the ion concentration = C_1_ (i.e. ion concentration following transportation to the other side of the membrane):

$j_{i}=\frac{u_{i}}{l}\times\Delta\Psi\times\frac{C_{o}-(C_{1}\times e^{-Z\times Y})}{1-e^{-Z\times Y}}$ (18)

Where Y is ΔΨ normalized by RT/F (equation 19) for cells at room temperature, 25°C, which equals 25.69 mV [6]:

$Y=\frac{\Delta\Psi\times F}{R\times T}$ (19)

Thus, substituting terms in equation 18 by the ones in equations 17, we obtain:

$j_{i}=\frac{D_{i}{\times Z}_{i}\times F}{R\times T\times l}\times\Delta\Psi\times\frac{C_{o}-(C_{1}\times e^{-Z\times Y})}{1-e^{-Z\times Y}}$ (20)

Moreover, equation 20 can be further simplified using the relationship in equation 19:

$j_{i}=\frac{D_{i}\times Z_{i}\times Y}{l} \times\frac{C_{o}-(C_{1}\times e^{-Z\times Y})}{1-e^{-Z\times Y}}$ (21)

Furthermore, the diffusion coefficient can be written in terms of the permeability of the ion across the membrane using the following equation:

$P_{i}= \frac{K_{i}{\times D}_{i}}{l}$ (22)

Where K_i_ is the water-membrane partition coefficient of the ion “i" and measures the solubility of the ion in lipids. This term is set to 1 for either a co-ion or a counterion since the pore size of an ion membrane transporter is generally large and the partition coefficient of an ion in a pore approaches 1 as the pore size increases [7].

Thus, by substituting equation 22 into equation 21, we obtain:

$j_{i}=P_{i}\times Y\times Z\times\frac{C_{o}-(C_{1}\times e^{-Z\times Y})}{1-e^{-Z\times Y}}$ (23)

Because equation 23 gives the ion flux over a single area in units of Molar per second, the rate is multiplied by the total lysosomal surface area, S, to determine the total ion flux, as follows:

$j_{i}={S\times P}_{i}\times Y\times Z \times\frac{C_{o}-(C_{1}\times e^{-Z\times Y})}{1-e^{-Z\times Y}}$ (24)

Accordingly, the total amount of free proton that can be transported from the lysosome to the cytoplasm, H_leak_, in units of Molar per second (which is further converted into molecules per second by multiplying it by Avogardo’s number, N_av_) follows the derivation of equation 24 and thus, can be written as:

$H_{\mathrm{leak}}= {(S\times P}_{H^{+}}\times Y\times Z\times\frac{{10}^{-pH_{L}} - \left( {10}^{-pH_{C}} \times e^{-Z\times Y} \right)}{1- e^{-Z\times Y}} )\times N_{\mathrm{av}}$ (25)

With pH^+^ being the proton permeability in units of cm/s, pH_L_ is the lysosomal luminal pH in units of pH units used to calculate the free lysosomal proton, 10^-pHL^, in units of Molar, pH_c_ is the cytoplasmic pH in units of pH units used to calculate the free cytoplasmic proton, 10^-pHC^, in units of Molar, Z, S, and Y are the same terms as previously described.

**Modeling the effect of membrane leaflet potentials on ions.** Using an equation derived from the previously defined GHK flux equation, the concentrations of the transported ions are further modified based on the surface membrane potential exposed to either the cytoplasm or the lysosomal compartment [6]. In the case where net current flow is zero, the GHK equation that defines the membrane potential, which in this case is also defined as Nernst potential, ΔΨ_i_, of a given ion “i”,is presented as:

${\Delta\Psi= \Delta\Psi}_{i}= \frac{-Z\times R\times T}{F}\times ln \frac{C_{i,0}}{C_{i,1}}$ (26)

With C_i_ being the concentration of the ion “i" in units of Molar and the position of the ion are denoted by the subscripts 0 and 1.

Thus, by inserting the intrinsic internal leaflet potential, Ψ_in_, in units of mV into equation 26, the concentration of a given ion at the membrane surface facing the lysosomal compartment is calculated according to the following relationship:

${\Delta\Psi}_{i,in}= \frac{-Z\times R\times T}{F}\times\ln\frac{C_{i,in}}{C_{i,L}}$ (27)

With C_i,in_ being the internal concentration of a given ion at the membrane surface facing the lysosomal compartment in units of Molar, and C_i,L_ is the concentration of the same ion in the non-membrane region of the lysosomal compartment in units of Molar.

Similarly, by inserting intrinsic external or outer leaflet potential, Ψ_out_, in units of mV into equation 26, the concentration of a given ion at the membrane surface facing the cytoplasmic compartment is calculated according to the following relationship:

${\Delta\Psi}_{i,out}= \frac{-Z\times R\times T}{F}\times ln \frac{C_{i,out}}{C_{i,C}}$ (28)

With C_i,out_ being the external concentration of a given ion at the membrane surface facing the cytoplasmic compartment in units of Molar, and C_i,c_ is the concentration of the same ion in the non-membrane region of the cytoplasmic compartment in units of Molar.

**Governing equations.** The aforementioned ion flux equations are used to generate a kinetic model to monitor the time-dependent changes in lysosomal proton and chloride molecules per second as described by equations 29 and 30:

$\frac{dH^{+}}{\mathrm{dt}}= H_{\mathrm{pump}}-{H_{ClC7}- H}_{\mathrm{leak}}$ (29)

$\frac{d\mathrm{Cl}^{-}}{\mathrm{dt}}=2\times N_{\mathrm{ClC}}\times J_{Cl, H_{ClC7}}\left( \Delta pH , \mathrm{Cl}_{L}, \mathrm{Cl}_{C}, \Delta\Psi\right)$ (30)

The coefficient “2” in equation 30 indicates that CLC7 transports chloride and proton across the lysosomal membrane in 2:1 stoichiometric ratio, which was used in published model [3] based on analysis of experimental studies [4].

We can further define the time-dependent change in pH by converting the total amount of lysosomal proton in units of molecules per second (equation 29) into units of Molar per second by dividing the terms in equation 29 by lysosomal volume and Avogadro’s number. Moreover, the Molar per second unit is converted into units of pH unit per second by dividing the entire term by the buffering capacity, β, which is represented in units of Molar per pH unit:

$\frac{\mathrm{dpH}}{\mathrm{dt}}= \frac{{(-H}_{\mathrm{pump}}+{H_{ClC7}+ H}_{\mathrm{leak}})}{V\times N_{\mathrm{av}}\times\beta}$ (31)

The lysosomal lumen buffering capacity of the Donnan particles entraps protons and thus, dictates lysosomal pH. It is experimentally measured by introducing a strong acid or base (in units of Molar) that can induce change of 1 pH unit. Thus, the physiological accumulation of weakly basic compounds, such as cationic amphiphilic and lysosomotropic drugs, will have insignificant effect on the buffering capacity, at least in theory, as the amount of proton they sequester from the lysosome will be fully released upon the hydration of the protonated drug, thereby maintaining mass-balance mediated physiological lysosomal pH. Although the buffering capacity varies with pH, for simplicity purpose, it was set to a constant according to literature values [3, 8].

**Numerical analysis.** The set of differential equations 29-31 was solved by numerical integration in Berkeley Madonna® using Rosenbrock stiff solver as a numerical integrator.

**References**

1. Fricke H. The Electric Capacity of Suspensions with Special Reference to Blood. J Gen Physiol. 1925;9(2):137-52.

2. Fenwick EM, Marty A, Neher E. Sodium and calcium channels in bovine chromaffin cells. J Physiol. 1982;331:599-635.

3. Ishida Y, Nayak S, Mindell JA, Grabe M. A model of lysosomal pH regulation. J Gen Physiol. 2013;141(6):705-20.

4. Graves AR, Curran PK, Smith CL, Mindell JA. The Cl-/H+ antiporter ClC-7 is the primary chloride permeation pathway in lysosomes. Nature. 2008;453(7196):788-92.

5. Weiss TF. Cellular Biophysics: Transport: MIT Press; 1996.

6. Hille B. Ionic Channels of Excitable Membranes. 2nd Edition. Sunderland, MA. : Sinauer Associates, Inc. ; 1992.

7. Buyukdagli S, Manghi M, Palmeri J. Variational approach for electrolyte solutions: from dielectric interfaces to charged nanopores. Phys Rev E Stat Nonlin Soft Matter Phys. 2010;81(4 Pt 1):041601.

8. Gekle M, Silbernagl S. Comparison of the buffer capacity of endocytotic vesicles, lysosomes and cytoplasm in cells derived from the proximal tubule of the kidney (opossum kidney cells). Pflugers Arch. 1995;429(3):452-4.
